# Protein Film Infrared Electrochemistry Demonstrated for Study of H_2_ Oxidation by a [NiFe] Hydrogenase

**DOI:** 10.3791/55858

**Published:** 2017-12-04

**Authors:** Philip A. Ash, Ricardo Hidalgo, Kylie A. Vincent

**Affiliations:** ^1^Department of Chemistry, University of Oxford, Inorganic Chemistry Laboratory

**Keywords:** Biochemistry, Issue 130, Protein Film Infrared Electrochemistry, PFIRE, hydrogenase, electrocatalysis, bioelectrocatalysis, fuel cell catalysis, protein film electrochemistry, *in situ* spectroscopy, vibrational spectroscopy, *in operando*, attenuated total reflectance, steady state kinetics, biophysics, redox proteins

## Abstract

Understanding the chemistry of redox proteins demands methods that provide precise control over redox centers within the protein. The technique of protein film electrochemistry, in which a protein is immobilized on an electrode surface such that the electrode replaces physiological electron donors or acceptors, has provided functional insight into the redox reactions of a range of different proteins. Full chemical understanding requires electrochemical control to be combined with other techniques that can add additional structural and mechanistic insight. Here we demonstrate a technique, protein film infrared electrochemistry, which combines protein film electrochemistry with infrared spectroscopic sampling of redox proteins. The technique uses a multiple-reflection attenuated total reflectance geometry to probe a redox protein immobilized on a high surface area carbon black electrode. Incorporation of this electrode into a flow cell allows solution pH or solute concentrations to be changed during measurements. This is particularly powerful in addressing redox enzymes, where rapid catalytic turnover can be sustained and controlled at the electrode allowing spectroscopic observation of long-lived intermediate species in the catalytic mechanism. We demonstrate the technique with experiments on* E. coli *hydrogenase 1 under turnover (H_2_ oxidation) and non-turnover conditions.

**Figure:**
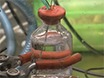


## Introduction

A key challenge in the study of protein function involves development of *in situ* methods that allow direct observation of proteins carrying out their physiological roles, either *in vivo *or using isolated protein samples. This requires the integration of control or triggering processes into experimental procedures and use of combined techniques that allow both the reactivity to be assessed, and individual chemical steps during protein function to be measured, simultaneously. In the case of redox proteins this often equates to combining electrochemical techniques, which precisely control the applied potential but provide no direct chemical information, with spectroscopic techniques that are sensitive to chemically-specific changes associated with protein function.^1^,^2^,^3^ Spectroelectrochemistry is a general term for a range of coupled electrochemical and spectroscopic methods that encompass a variety of spectroscopic techniques and levels of electrochemical control. Many proteins can exchange electrons with artificial, soluble electron donors and acceptors and this has been exploited in studies which use small molecules to mediate electron transfer, including coupling with UV-visible,^4^,^5^,^6^,^7^ magnetic circular dichroism^8^ and infrared^5^,^9^,^10^,^11^,^12^,^13^,^14^ (IR) spectroscopies. In a limited number of cases it has proved possible to exploit unmediated, diffusion-controlled electron exchange between proteins and electrodes.^15^,^16^

For catalytic reactions carried out by redox enzymes, solution electrochemistry approaches present a clear disadvantage. Diffusion-controlled electron transfer *via* redox mediators in solution is likely to become rate limiting. Kinetic and mechanistic information about the enzyme may be lost, or at least become difficult to deconvolute from diffusion artefacts resulting from the experimental method. Direct, unmediated electrochemical control is therefore an important tool for the study of redox proteins and enzymes. The technique of protein film electrochemistry (PFE) employs electrode-immobilized redox proteins, in such a way that electrons are transferred directly to or from the redox cofactors within the protein as the electrode is polarized at a series of potentials.^17^,^18^,^19^ PFE is of particular value for the study of oxidation or reduction reactions catalyzed by redox enzymes, as interfacial electron transfer can be achieved at a very high rate. For example, the electrocatalytic turnover rate of the nickel-iron ([NiFe]) hydrogenase from *Allochromatium vinosum* was measured by PFE as *ca* 1,000 - 10,000 s^−1^ for H_2_ oxidation.^20^ The electrode potential acts as a trigger to turn catalysis 'on' or 'off', and the electrocatalytic current reports on enzyme activity. PFE is therefore a valuable method for analyzing the reactivity of complex enzymes that depend intimately on potential, such as reactions of the di-iron active site of [FeFe]-hydrogenases with CO and O_2_,^21^ or potential-induced inactivation reactions of hydrogenases,^22^ carbon monoxide dehydrogenase,^23^ and other complex redox enzymes.^24^

The principal barrier to combining spectroscopic techniques with the direct electrochemical control afforded by PFE arises from the low surface coverage of redox enzymes, on the order of 1-2 pmol cm^-2^ for the [NiFe] hydrogenase from *A. vinosum*,^20^ relative to *in situ* surface science studies of small molecule adsorbates on bulk metal electrodes. This presents a challenge for the sensitivity of the spectroscopic measurement. Several spectroelectrochemical methods have been reported for studying immobilized redox proteins at a range of different electrodes: UV-visible spectroscopy at transparent metal oxide electrodes;^25^,^26^,^27^ fluorescence spectroscopy at gold electrodes;^28^,^29^ surface enhanced infrared absorption (SEIRA) spectroscopy at gold electrodes;^30^,^31^,^32^,^33^ and surface enhanced Raman spectroscopic techniques, principally at silver electrodes.^34^,^35^

Here we describe a method for coupling PFE with IR spectroscopy, in a technique known as protein film infrared electrochemistry (PFIRE).^36^ The PFIRE method studies redox enzymes immobilized on a high surface area carbon working electrode in conjunction with an attenuated total reflectance IR (ATR-IR) geometry, exploiting the ease of adsorption of a range of proteins onto carbon surfaces. IR spectroscopy is useful in studies of redox enzymes and proteins as many small molecules, ligands and cofactors have diagnostic absorbances that can be used to assess reactivity, binding, inhibition and redox state. Examples include binding of NO to iron-sulfur centers,^37^ study of flavoproteins,^38^,^39^,^40^ small molecule binding to heme centers *etc*.^41^ The ATR-IR geometry allows construction of an optimized three-electrode (spectro)electrochemical cell^42^ and therefore provides excellent electrochemical control. Solution resistance and potential drift are minimized by placing a reference electrode close to the working electrode. High surface area counter electrodes are used that are compatible with high electrocatalytic currents produced by fast enzyme turnover at the working electrode. Flow of solution through the spectroelectrochemical cell allows facile control over the concentration of substrates, inhibitors and pH.^36^,^43^,^44^ The PFIRE method therefore allows IR spectra to be recorded *in situ* during sustained enzyme electrocatalysis.^36^,^44^ PFIRE is also capable of providing chemical information in the absence of catalytic current,^43^ in contrast to PFE where it can be difficult to extract information from non-catalytic processes in redox enzymes.^45^,^46^

We have demonstrated the PFIRE method for study of electrocatalytic H_2_ oxidation by [NiFe] hydrogenases, which contain intrinsic CO and CN ligands coordinated to Fe at a bimetallic active site.^36^,^43^,^44^ [NiFe] hydrogenases are therefore particularly well suited to study by PFIRE. The PFIRE method provides information about the species that are present during steady-state turnover, and therefore provides crucial mechanistic insight in addition to the wealth of literature on IR spectroscopy of hydrogenases without experimental control over turnover.^47^,^48^ Dyer and co-workers have employed time-resolved IR methods to study of NiFe hydrogenases,^49^,^50^,^51^ using a light trigger to either apply a small negative potential step (*via* use of solution mediators and a 'caged' electron source) or photolyse a bound hydride. Although the PFIRE method cannot, at present, provide time resolution to match these measurements,^40^ it does allow study of both reductive and oxidative catalytic processes, accessed at a range of well-defined potentials and free from mass transport limitations.

The PFIRE method is distinct from SEIRA studies of redox proteins, which also use an ATR-IR geometry and employ a nanoscale-roughened metal electrode to enhance the IR absorbance of molecules adsorbed on the electrode surface.^30^ SEIRA is an extremely valuable technique for studying membrane proteins, in particular adsorbed on or within mimetic membrane architectures,^32^ but the need for a metal electrode can limit the substrate and inhibitor scope due to reactivity of the electrode support towards small molecules such as CO, CN^-^, CO_2_* etc.* Proton reduction and self-assembled monolayer desorption can be problematic on metal surfaces at very negative potentials,^1^,^52^ although enzyme electrocatalysis on unprotected metal electrodes has been reported.^53^,^54^ A disadvantage of PFIRE relative to SEIRA is the relative difficulty of incorporating membrane proteins in native or mimetic membrane architectures. However, the relative chemical inertness of the carbon electrodes to competing small molecule activation reactions makes PFIRE an excellent technique for studying enzyme electrocatalysis, particularly in the low-potential domain relevant to biological redox processes such as proton reduction by hydrogenases.^1^,^43^

The aim of this article is to introduce the PFIRE method as a technique for studying electrode-immobilized redox proteins, using NiFe hydrogenase 1 (Hyd1) from *Escherichia coli *as an example. Considerations of sample preparation, the requirement for good substrate flow, and data handling are discussed. PFIRE is a broadly applicable technique, well-suited to studying any redox protein (with characteristic IR absorbances) that can be adsorbed onto carbon electrodes, either directly or using surface modification, such that it can exchange electrons with the electrode.

## Protocol

### 1. Recreating the Internal Sample Compartment of an FTIR Spectrometer Inside an Anaerobic, Dry Glovebox and General Experimental Requirements

1. Use a commercial FTIR spectrometer equipped with an external mercury cadmium telluride (MCT) detector and ATR accessory. Purge the body of the spectrometer with dry air or dinitrogen, or alternatively use a spectrometer with an evacuated optical bench.
2. Place the spectrometer onto a stable, vibration-free table at the same height as an anaerobic (<1 ppm O_2_), dry (dew point < -75 °C) glovebox. Divert the IR beam of the spectrometer into the glovebox, through an IR transparent window that is large enough to accommodate the defocused beam. NOTE: It is important that the space between the glovebox and spectrometer is also purged or evacuated, especially if a hygroscopic window material, such as NaCl or KBr for example, is used.
3. **Replicate the internal sample compartment of the spectrometer inside the glovebox.**
  1. Direct the beam inside the glovebox onto an off-axis ellipsoidal mirror, ideally with the same focal length as the internal focusing mirror of the spectrometer. NOTE: It is useful to have two additional plane mirrors placed before this focusing mirror, in order to allow both the height and the direction of the IR beam to be adjusted inside the glovebox; this will offset any inaccuracy in initial placement of the spectrometer.
  2. Place an external MCT detector, complete with a suitable focusing optic (such as a short focal length ZnSe lens, or off-axis parabolic mirror, with a short focal length) inside the glovebox, positioned such as to replicate the dimensions of the internal sample compartment of the spectrometer.
  3. Adjust the 'focus' of the IR beam to be approximately equidistant between the focusing mirror and the MCT detector.
4. Cool the dewar of the external MCT detector with liquid nitrogen.
5. Mount the ATR accessory into the glovebox sample compartment. Align the input and output focusing optics and ATR accessory to achieve maximum throughput to the MCT detector. If necessary, use an aperture or other suitable attenuation (such as a wire grid) to prevent oversaturation of the detector signal. NOTE: For the data presented here, a five-reflection ATR accessory with all reflective optics and a removable trapezoidal Si internal reflection element (IRE) is used (Crystal GmbH, dimensions *ca* 5 × 8 × 1 mm^3^, face angle 39.5°). The IRE is mounted in a removable baseplate machined from polyether ether ketone (PEEK).
6. Use a glovebox to house the recreated sample compartment with suitable feedthroughs to allow connection to an externally-housed potentiostat (gas tight BNC connections are useful for this purpose), gas access, and cable(s) to transfer the signal from the MCT detector. It is important to retain any shielding on the detector cable to avoid introducing noise or interference to the measurements.
7. Place a peristaltic pump, capable of flow rates greater than 60 mL/min inside the glovebox. A schematic view of the spectrometer, ATR accessory, peristaltic pump and potentiostat setup is shown in Figure 1.
8. Construct a spectroelectrochemical cell, such as that shown schematically in Figure 2, that seals onto the ATR accessory. The cell should contain a high surface area counter electrode (Pt wire or gauze), a connection for the working electrode (carbon rod) and a miniature reference electrode. The cell should contain at least two further ports as inlet and outlet for rapid flow of solutions through the cell. NOTE: A miniature saturated calomel reference electrode is ideal for this purpose.^36^

### 2. Preparation of Carbon Black Particles Modified with *E. coli *Hydrogenase 1

1. In a 'wet' anaerobic glovebox, mix 20 mg of high surface area carbon black particles (>1,000 m^3^/g) with 1 mL ultrahigh purity water (resistivity >18 MΩ cm) in a microcentrifuge tube. Disperse the particles by low-power sonication (<100 W) for at least 15 min, or until the dispersion is uniform and does not sediment within 1 h, to give a carbon black dispersion with a loading of approximately 20 mg/mL.
2. **Take an aliquot of *E. coli *hydrogenase 1 (Hyd1, approximately 50 µL, prepared according to a published procedure^55^) at a concentration of ~7 mg protein/mL and exchange it into a low ionic strength buffer at a pH close to the isoelectric point (for example potassium phosphate, 15 mM, pH 5.8, no additional salt). To achieve best results, use an enzyme preparation that is as active as possible. Perform the buffer exchange by concentration and dilution, in a centrifugal filter device with an appropriate molecular weight cutoff (50 kDa works well for for Hyd1).**
  1. Add the Hyd1 to the filter device.
  2. Dilute with approximately 450 µL of exchange buffer.
  3. Reconcentrate to a volume of 50 µL using mild centrifugation (< ~2700 × *g*) to prevent irreversible precipitation.
  4. Repeat steps 2.2.2 and 2.2.3 until buffer exchange is complete (typically within ~5 cycles).
3. Mix a 5 µL volume of 20 mg/mL carbon black dispersion (prepared in 2.1) with the 50 µL aliquot of buffer-exchanged Hyd1. Store the enzyme-particle mixture in a refrigerator (at 0 °C) overnight to allow the enzyme to adsorb. It may be necessary to shake the mixture occasionally to ensure the particles remain dispersed.
4. Wash the Hyd1-modified particles with low ionic strength buffer (potassium phosphate, 15 mM, pH 5.8, no additional salt) by successive sedimentation and re-dispersion cycles in a microcentrifuge (~2,700 × *g*). Repeat this process 3-5 times to remove non-adsorbed enzyme. NOTE: Before the first wash the supernatant should be virtually colorless, indicating good adsorption of enzyme.
5. Concentrate the particles to a final volume of ~5 µL, to give a final loading of ~20 mg/mL of enzyme-modified particles. NOTE: The Hyd1-modified particles can be stored at 4 °C for up to two weeks if they remain hydrated; this is best achieved by storing particles diluted with ~50 µL of ultrahigh purity water.

### 3. Preparation for PFIRE Measurements on *E. coli *Hydrogenase 1

1. Clean the Si IRE by low-powered sonication (< 100 W), first in H_2_SO_4_ (< 90 % w/w) for ~15 min, and then in HNO_3_ (70 % w/w) for up to 1 hr. This cleaning procedure should lead to a hydrophilic IRE surface. If further cleaning is required the IRE can be placed in piranha solution (a 1:3 ratio of H_2_O_2_:H_2_SO_4_) to oxidize any remaining organic material. NOTE: Harsh acidic cleaning methods may not be suitable for use with all IRE materials. Piranha solution is highly corrosive and a strong oxidant, surfaces should be reasonably clean before use of piranha solution and care should be taken during preparation and use of piranha solution due to the exothermic nature of the process - always add H_2_O_2_ **slowly** to H_2_SO_4_ and refer to the appropriate safety procedures for preparation, use and disposal.
2. Rinse the clean IRE in ultrahigh purity water, and dry under a stream of dry nitrogen gas. Carry out all prism handling using clean tweezers to avoid contamination.
3. Seal the IRE into the ATR accessory baseplate using a thin strip of electrical-grade silicone sealant. Take care to restrict the sealant to the edges of the IRE. Allow the sealant to dry fully.
4. Transfer the ATR accessory baseplate to the spectrometer glovebox and mount it onto the ATR accessory. Measure a reference (background) spectrum between 4000 - 1,000 cm^-1^ at 4 cm^-1^ resolution, using the standard rapid scan mode of the spectrometer and 1024 averaged interferograms (measurement time ~3 - 5 min). Use this spectrum as an input to allow computation of absorbance spectra later in the experiment. NOTE: Due to the small absorbance expected for the Hyd1 active site it is necessary to collect spectra with high signal to noise ratios.
5. Transfer the ATR accessory baseplate to the 'wet' glovebox which contains the pre-prepared Hyd1-modified particle dispersion (section 2). Drop-cast a 1 µL aliquot of the particle dispersion onto the large face of the IRE, and spread them evenly across the surface. Note: Do not allow the particles to become completely dry on the IRE.
6. Cut a piece of carbon paper, such as that used as gas diffusion layer material in fuel cells, to a size ~0.1 mm smaller than the surface dimensions of the IRE (*i.e. ca.* 8.2 × 4.9 mm^2^). The surface area of the carbon paper should be large enough to cover the drop-cast Hyd1-modified particles, but not so large as to overlap the silicone sealant used to secure the IRE. Soak the carbon paper in water, and gently place it over the top of the particle film. The carbon paper will provide a good electrical connection across the whole particle film. NOTE: It is beneficial to prepare several pieces of carbon paper in advance, and store them pre-soaked in ultrahigh purity water inside the 'wet' anaerobic glovebox.
7. Mount the spectroelectrochemical cell (described in 1.7 and shown schematically in Figure 2) over the IRE, secure it into the baseplate with screws. Add ~200 µL of the experimental buffer to keep the enzyme hydrated. A mixed buffer system, capable of buffering over a wide pH range, is useful for studies on NiFe hydrogenases:^56^ sodium acetate, 2-[*N*'-morpholino]ethane-sulfonic acid (MES), *N*'-[2-hydroxyethyl]piperazine-*N*'-[2-ethane-sulfonic acid] (HEPES),* N*'-tris[hydroxymethyl]methyl-3-amino-propane-sulfonic acid (TAPS), and 2-[*N*'-cyclohexylamino]ethane-sulfonic acid (CHES), with each component at a final concentration of 15 mM and containing 0.1 M NaCl as supporting electrolyte, with pH adjusted to pH 6 using concentrated NaOH and HCl. NOTE: The working electrode connection should protrude slightly below the plane of the spectroelectrochemical cell top (~0.1 mm) to ensure good electronic connection to the carbon paper (Figure 2).
8. Connect the solution inlet and outlet of the spectroelectrochemical cell to a flow system containing peristaltic pump tubing and a vial of the experimental buffer. Transfer the assembled cell to the 'dry' glovebox containing the spectrometer.
9. Mount the spectroelectrochemical cell assembly onto the ATR accessory. Connect the working, counter and reference electrodes to the potentiostat. Connect the peristaltic pump tubing to the pump.
10. Record an absorbance spectrum with 1024 averaged interferograms at 4 cm^-1^ resolution over a spectral range of 4,000 - 1,000 cm^-1^, using the spectrum collected in 3.4 as a reference/background. At this point the spectrum should contain significant (>100 mO.D.) amide II bands at ~1,540 cm^-1^ and active site bands of Hyd1 should be evident in the spectral region 1,850 - 2,150 cm^-1^, largely in oxidized, inactive states (Figure 3).

### 4. Activation of *E. coli *Hydrogenase 1 and Testing the Spectroelectrochemical Cell

1. Apply a reducing potential (−0.8 V *vs* SCE) to the Hyd1-modified particle film. Saturate the experimental buffer with H_2_ and flow buffer slowly through the spectroelectrochemical cell. Leave the sample overnight to fully activate the Hyd1. NOTE: It is important to use anaerobic H_2_, N_2 _*etc.*, and so all anaerobic gases should be passed through an O_2_ filter.
2. Record an absorbance spectrum of the sample after activation. The *ν*_CO_ and *v*_CN_ bands of the active site should now show a distribution of reduced, 'active' states. This is most easily observed through the use of a difference spectrum, relative to the spectrum recorded in 3.10 (Figure 4).
3. Test the electrical connection of the spectroelectrochemical cell. To do this, saturate the experimental buffer with N_2_ gas. Apply a sequence of oxidizing (0 V *vs* SCE) and reducing (−0.8 V *vs *SCE) potentials (of *ca* 30 min duration) to the Hyd1-modified particle film, and record an absorbance spectrum at each. The Hyd1 should become rapidly oxidized and reduced, and if the particle film is well connected 100 % of the sample should respond to the applied potential.
4. Set an appropriate flow rate of the experimental buffer. To do this, apply a reducing potential (−0.8 V *vs *SCE) to the sample, and saturate the experimental buffer with H_2_. Record a series of cyclic voltammograms between -0.707 - 0.039 V *vs* SCE at a scan rate of 10 mV/s. Gradually increase the flow rate of H_2_-saturated buffer between voltammograms until the catalytic waveshape resembles that at a planar rotating disc electrode^55^ and the maximum current is independent of flow rate (Figure 5). NOTE: The solubility limit of H_2_ in water is ~0.8 mM at 293 K and 1 bar.
5. As the sample is now ready for PFIRE measurements, collect spectra at a range of potentials, under a range of solution conditions (pH, temperature, H_2_ concentration *etc.*). Record all electrochemical data using the potentiostat software as it is important to be able to correlate spectroscopic and electrochemical data, especially when studying electrocatalytic processes such as H_2_ oxidation by Hyd1.

### 5. Spectroscopic Data Handling

1. Confirm that the active site has not been permanently altered during the course of the measurements by recording spectra at 0 V and −0.8 V *vs *SCE at the end of the experiment. These should be identical to the spectra recorded in 4.3, and no loss of active site should be observed during the measurement (Figure 6).
2. Export absolute absorbance spectra from the spectrometer software in a suitable format (.csv, ASCII, 'matlab', Jcamp *etc.*) for processing using software such as Origin or Matlab.
3. **Baseline correct the data, using the process illustrated in Figure 7.**
  1. Take the second derivative of each absorbance spectrum in the range 1,800 - 2,150 cm^-1^, in order to identify small (<1 mO.D.) active site bands against the highly-curved water background.
  2. Place baseline marker points on the original absorbance spectrum, and set the points to 'snap' to the experimental spectrum.
  3. Fit a baseline through the points using an interpolated cubic spline function or a polynomial function. Subtract this baseline function from the experimental data.

## Representative Results

Figure 1 shows a schematic representation of the experimental arrangement of the spectrometer, glovebox, ATR accessory, potentiostat and gas flow system used for PFIRE measurements. Figure 2 shows a representative drawing of the spectroelectrochemical cell.

Figure 3 shows absorbance spectra of drop-cast Hyd1-modified particles, with the experimental buffer (a mixed buffer system, described in 3.7, pH 6.0) flowing through the spectroelectrochemical cell. The surface coverage of Hyd1 is particularly high in the example shown in Figure 3, with an amide II band intensity of ~235 mO.D. and minimal 'bulk' water as evidenced by the magnitude of the O-H stretching region (~3,000 - 3,600 cm^-1^) relative to the band at ~1,640 cm^-1^, which is a convolution of the amide I band of Hyd1 and the water H-O-H bend. Additional bands due to the protein can be seen in the C-H stretching region (ca 2900 cm^-1^). The broad band centered around 2,100 cm^-1^ is a combination band of the H-O-H bending vibration with a set of lower energy libration bands, which are restricted rotations of H_2_O molecules due to the hydrogen bonding network in liquid water. The *ν*_CO_ band of the oxidized, inactive, Ni-B state of the active site is clearly evident at 1,943 cm^-1^, even without baseline correction, and *ν*_CN_ features are clearly visible between 2,050-2,100 cm^-1^. At high Hyd1 coverages, much of the microporous structure of the carbon black film^57^ becomes blocked by enzyme and therefore the 'bulk' water concentration is lowered during PFIRE measurements.

The spectra in Figure 3 show that, before activation, Hyd1 films contain Hyd1 in oxidized, inactive states. Activation overnight at −0.8 V vs SCE under a H_2_ atmosphere leads to formation of reduced, catalytically active states as demonstrated in Figure 4 which shows an activated (reduced) *minus *as-prepared (oxidized) difference spectrum of Hyd1. Difference spectra of Hyd1 can be most clearly interpreted using the* ν*_CO_ region. Each unique state of the active site has only one CO band compared to two CN bands and therefore the *ν*_CN_ region is intrinsically more complicated, with many overlapping bands. The difference spectrum in Figure 4 shows that activation leads to loss (negative absorption bands) of oxidized, inactive Ni-B and a small amount of Ni-SI (the most oxidized 'active' state) that was present in the as-prepared Hyd1 film. These are replaced by 'active' states of Hyd1; Ni-C, Ni-R and Ni-L. Note that there are two forms of both the Ni-R and Ni-L states, as evidenced by the two *ν*_CO_ bands observed for these species in Figure 4. The observation of multiple Ni-R and Ni-L states is in agreement with other NiFe hydrogenases.^48^,^58^,^59^

A key verification of the PFIRE method is that the cyclic voltammograms recorded of Hyd1 inside the spectroelectrochemical flow cell show similar catalytic waveshapes to those recorded on a planar rotating disc electrode.^55^ In practice this means that the mass transport of substrate (H_2_) and product (H^+^) to/from immobilized Hyd1 in the spectroelectrochemical cell is efficient at the flow rates used during PFIRE measurements. The effect of flow rate on the catalytic waveshape is shown in Figure 5, which shows successive voltammograms recorded under a H_2_ atmosphere (1 bar) as the solution flow rate through the spectroelectrochemical cell is increased. In all cases the overpotential for H_2_ oxidation by Hyd1 is identical (red shaded rectangle), but the extent of oxidative inactivation (hysteresis between the current during the oxidative and reductive sweeps at potentials above *ca *0 V *vs* SCE) and the maximum H_2_ oxidation current are dependent upon the solution flow rate. At flow rates above 52 mL/min (light grey voltammogram) the catalytic waveshape is insensitive to further flow rate increases.

Figure 6 compares the relative intensities of the *ν*CO band of the Ni-B state after initial activation and anaerobic re-oxidation at 0 V *vs* SCE (under a Ar atmosphere, as described in 4.3) and after anaerobic oxidative inactivation under an Ar atmosphere at 0 V *vs *SCE after 48 hr of continuous experiments (as described in 5.1). No loss of active site band intensity is observed during the measurement, and all the Hyd1 sample responds to the applied potentials.

Figure 7 demonstrates the baseline correction procedure used throughout this work. The absolute spectrum of the Hyd1 sample in the active site region (Figure 7**a**) contains significant curvature due to water. In fact, the requirement to use water as a solvent is a problem for most applications of IR spectroscopy in the life sciences. The second derivative of the absolute spectrum (Figure 7**b**), calculated using Origin software with Savitsky-Golay smoothing over a 9 point window, can be used to identify sharp bands of the Hyd1 active site against the curved background. The identification of approximate peak positions using a second derivative spectrum allows baseline anchor points to be placed in regions of the absolute spectrum that are free of active site bands (circles in Figure 7**a**). A cubic spline function is then fitted through these anchor points to create a baseline function that can then be subtracted from the absolute spectrum to give a baseline-corrected spectrum containing only peaks arising from the Hyd1 active site (Figure 7**c**).

Figure 8 shows the results of a PFIRE measurement on Hyd1, under both non-turnover (Ar atmosphere) and turnover (H_2_ atmosphere) conditions at a range of potentials.^36^ The current-time traces (Figure 8**a**) report on the catalytic current at each applied potential and remain at close to zero current under non-turnover conditions (Ar atmosphere). The partial spectroelectrochemical redox titration in Figure 8**b** therefore reports on the equilibrium redox behavior of the active site, showing the distribution of states expected at each potential in the absence of catalytic turnover. The spectra in Figure 8**c** were recorded under turnover conditions (under a H_2_ atmosphere) and therefore represent the steady-state distribution of active site states present during catalytic H_2_ oxidation by Hyd1. That steady-state conditions have been achieved is confirmed by the fact that the catalytic H_2_ oxidation current (Figure 8**a**, H_2_) remains constant as a function of time at −0.199 V and −0.074 V *vs* SHE; the monotonic decay in current at +0.356 V *vs *SHE is due to the well-known anaerobic oxidative inactivation of Hyd1.^55^ The distribution of active site states is clearly different under Ar and H_2_ at all potentials where Hyd1 performs catalysis (Figure 8**b**, spectra at −0.199, −0.074 and +0.356 V *vs *SHE). The spectra recorded under Ar and H_2_ are virtually identical at −0.594 V *vs* SHE, however, and this represents an important test of experimental consistency; Hyd1 does not reduce H^+^ at a significant rate at pH 6.0 (the current in Figure 8**a** under both Ar and H_2_ is close to zero), and the spectra at −0.594 V are therefore expected to be the same.

Figure 9 demonstrates the anaerobic oxidative inactivation of Hyd1 *via* the formation of Ni-B from Ni-SI during H_2_ oxidation at +0.356 V.^36^ Spectra were recorded during the grey time intervals noted on the current-time trace in Figure 9**a**. At −0.074 V Hyd1 does not undergo oxidative inactivation, and the distribution of active site states remains constant throughout the entire potential step. This is demonstrated by the spectra in Figure 9**b**_i_, which reports the absolute baseline-corrected spectrum at the beginning of the −0.074 potential step, and Figure 9**b**_ii_ which was recorded at a later time during the potential step and is reported as a difference spectrum relative to Figure 9**b**_i_. The spectra in Figure 9**b**_iii_ and **b_iv_** are also reported as difference spectra relative to **Figure 9b_i_**, and show gradual conversion of Ni-SI to Ni-B during high potential inactivation, consistent with the monotonic decrease in current shown at +0.356 V in Figure 9**a**.

Spectra recorded at a range of solution pH give insight into the proton transfer steps during the Hyd1 catalytic cycle.^43^ Figure 10**a** shows PFIRE spectra recorded on the same Hyd1 film at pH 3.0 and pH 9.0, using solution flow in the spectroelectrochemical cell to exchange the experimental buffer. The relative concentrations of the Ni-C and Ni-L states are clearly different in these two spectra. By varying the applied potential under non-turnover conditions the potential dependence of the NiC and Ni-L states can be determined rapidly at a range of pH values (Figure 10**b**), and both states are found to be isopotential over a wide pH range. (Note that the peak absorbances in Figure 10**b** show only the Ni-C and Ni-L states for clarity, full redox titrations of Hyd1 have been reported by Hidalgo *et al.*)^36^ A pH titration of the concentrations of Ni-C and Ni-L can then be extracted, taking the peak absorbance at potentials where the total Ni-C and Ni-L concentration is at its maximum at each pH (Figure 10**c**). In this way, and in conjunction with EPR data, a pH equilibrium between the Ni-C and Ni-L states was identified.^43^

**Figure:**
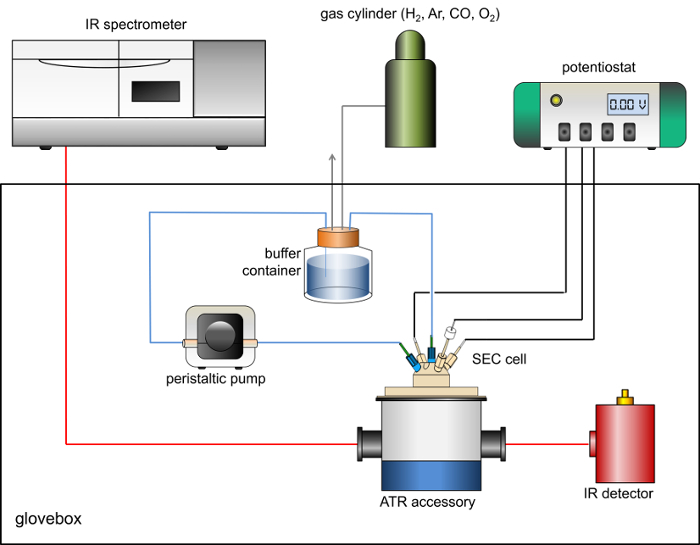

Figure 1: **Schematic of the arrangement of IR spectrometer, anaerobic glovebox, ATR accessory, MCT detector, gas flow system and potentiostat used for PFIRE measurements.** Please click here to view a larger version of this figure.

**Figure:**
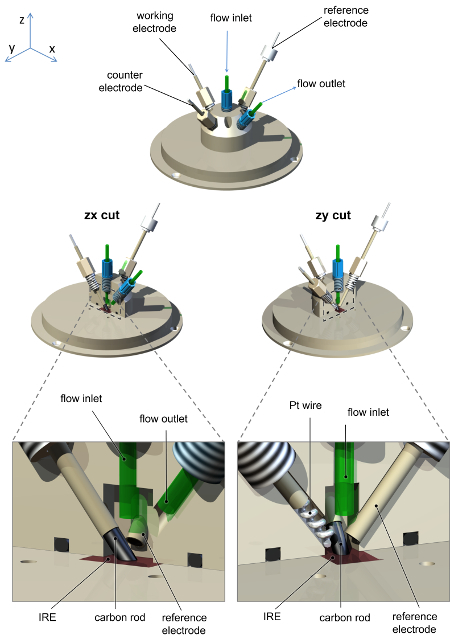

Figure 2:** Schematic diagram of the spectroelectrochemical cell used for PFIRE measurements, showing the arrangement of electrodes and solution inlet/outlet connections. **The cell and baseplate are machined from polyether ether ketone (PEEK), with screw holes for a carbon rod working electrode connection, Pt wire counter electrode, saturated calomel reference electrode, and solution inlet and outlet. The saturated calomel reference electrode construction is as previously reported.^36^Please click here to view a larger version of this figure.

**Figure:**
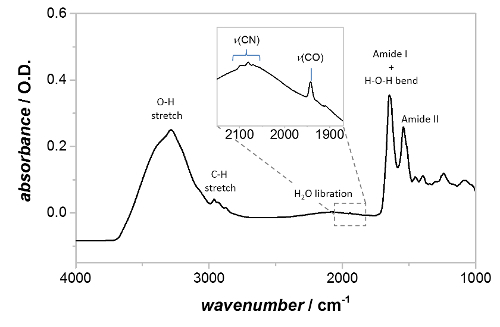

**Figure 3: Absorbance spectrum of Hyd1-modified carbon black particles, deposited onto the IRE and rehydrated with buffer. **The positions of the amide I band, amide II band, and Hyd1 active site region are shown, along with additional features due to C-H stretching vibrations and solvent water. The inset shows a magnified view of the active site region, with the *v*_CO_ and *v*_CN_ bands labeled. ‘As-prepared’ particles contain Hyd1 mainly in the oxidized, inactive Ni-B state. Please click here to view a larger version of this figure.

**Figure:**
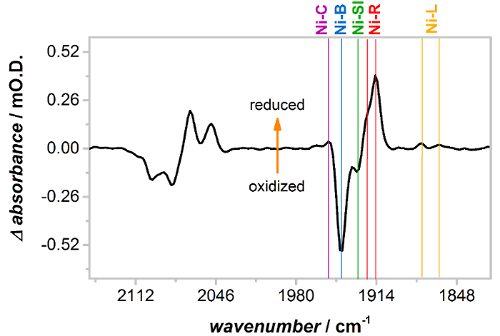

Figure 4**: Activation of Hyd1 at −0.8 V *vs* SCE under a H_2_ atmosphere, presented as a reduced *minus *oxidized difference spectrum. **Upon low potential activation oxidized, inactive Ni-B (and a small concentration of Ni-SI) converts to more reduced, active states Ni-C, Ni-R and Ni-L. Note that Hyd1 has two distinct sub-states of Ni-L and Ni-R. Please click here to view a larger version of this figure.

**Figure:**
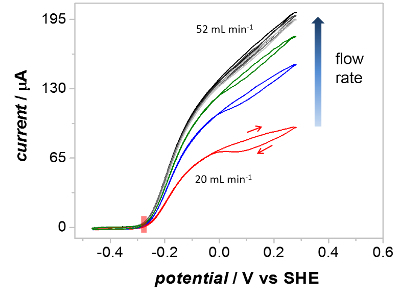

Figure 5**: The effect of solution flow rate on the waveshape of catalytic cyclic voltammograms recorded in the spectroelectrochemical cell. **Voltammograms were recorded at increasing flow rates of H_2_-saturated buffer as indicated. At a flow rate of 20 mL/min (red) the voltammogram shows significant inactivation above 0 V *vs* SHE on the forward scan. At flow rates above 52 mL/min the extent of inactivation is vastly lower and the current is independent of flow rate at all potentials. Other parameters: 1 bar H_2_, 10 mV/s scan rate. Please click here to view a larger version of this figure.

**Figure:**
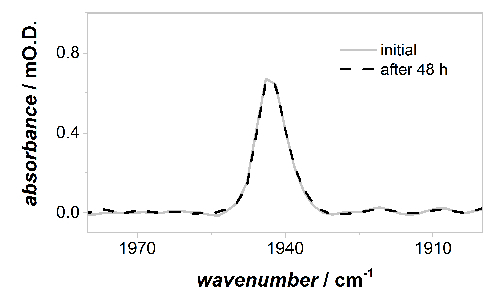

Figure 6**: Baseline corrected IR spectra in the active site *ν*CO region of oxidized, inactive Ni-B state at 0 V *vs *SCE. **There is no measurable loss of active site intensity during 48 h of continuous PFIRE measurements, and therefore Hyd1 is adsorbed robustly on the carbon black particles. Please click here to view a larger version of this figure.

**Figure:**
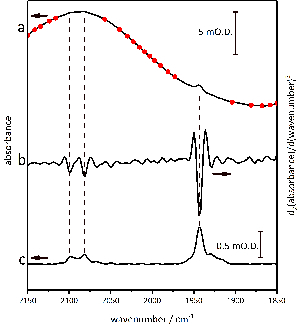

Figure 7:** Details of the baseline correction procedures used for data handling. **Baseline anchor points are placed onto the absolute absorbance spectrum in the active site region (**a**), taking care to avoid any *ν*_CO_ and *ν*_CN_ peaks identified from a second derivative analysis (**b**). The resulting baseline corrected spectrum is shown in (**c**). Please click here to view a larger version of this figure.

**Figure:**
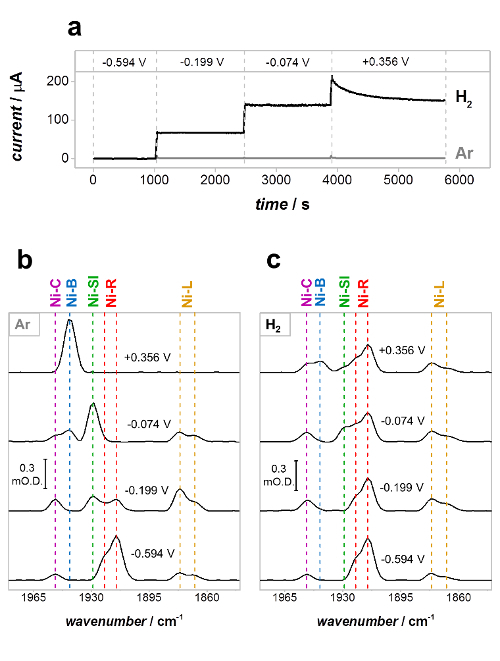

Figure 8:** PFIRE measurements on Hyd1 under non-turnover (Ar) and turnover (H_2_) conditions. **(**a**) Current-time traces of Hyd1 in the spectroelectrochemical cell in Ar-saturated (gray) and H_2_-saturated (black) buffer; (**b**), (**c**) PFIRE spectra showing the *ν*_CO_ region at each potential under Ar (b) and H_2_ (c). Potentials quoted in V *vs* SHE. Reproduced with permission from Hidalgo *et al.*^35^ Please click here to view a larger version of this figure.

**Figure:**
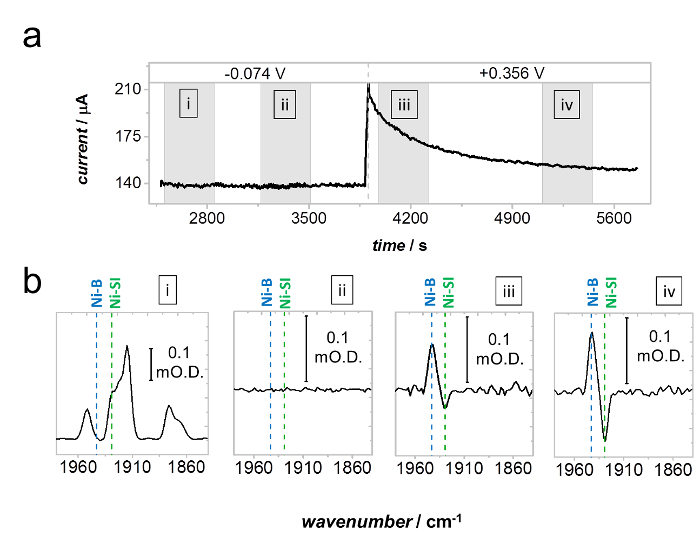

Figure 9:** Anaerobic inactivation of Hyd1 *via *formation of Ni-B from Ni-SI. **(**a**) Current-time trace under a H_2_ atmosphere, showing a stable electrocatalytic current at −0.074 V and slow anaerobic inactivation (monotonic decrease in current) at +0.356 V *vs *SHE. (**b**) Spectra recorded during the gray shaded regions marked in (a). Spectrum b_i_ is a baseline corrected spectrum recorded at the beginning of the −0.074 V potential step. Spectrum b_ii_, recorded at a later time during the −0.074 V step, is reported as a difference spectrum relative to b_i_ and shows that no change in distribution of active site states occurs, consistent with the stability of the potential at −0.074 V. Spectra b_iii_ and b_iv_ are also reported as difference spectra relative to b_i_ and show gradual conversion of Ni-SI to Ni-B during anaerobic inactivation at +0.356 V *vs* SHE. Reproduced with permission from Hidalgo *et al.* 35 Please click here to view a larger version of this figure.

**Figure:**
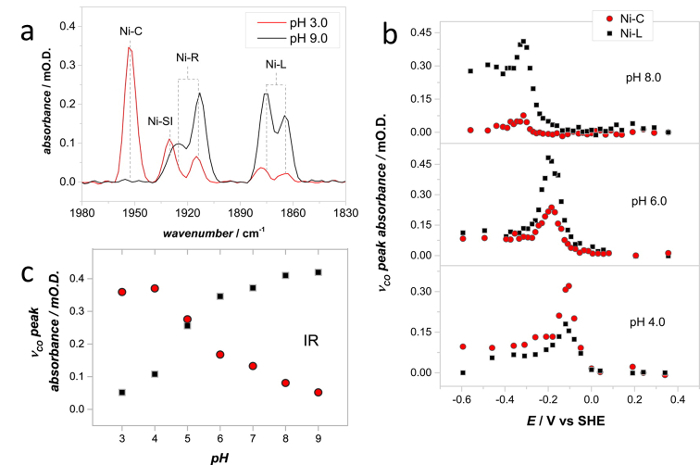

Figure 10**: Spectra recorded at a range of solution pH give insight into the proton transfer steps during the Hyd1 catalytic cycle. **(**a**) IR spectra showing the *ν*_CO_ region of Hyd1, recorded at pH 3.0 (-54 mV *vs* SHE) and pH 9.0 (-334 mV *vs* SHE). (**b**) Spectroelectrochemical titrations were carried out to determine the potential at which the Ni-C and Ni-L concentrations are at a maximum at a range of solution pH values. Only the Ni-C and Ni-L concentrations are shown for clarity, for a full spectroelectrochemical titration of Hyd1 see Hidalgo *et al.*^36^ (**c**) pH dependence of the relative concentration of Ni-C and Ni-L, as determined from a series of experiments such as those shown in (b). Spectra were recorded at 20 °C. Adapted with permission from Murphy *et al.*^42^ Please click here to view a larger version of this figure.

## Discussion

PFIRE is a broadly applicable IR spectroscopic technique for addressing electrode-immobilized redox proteins. In particular, electrocatalytic reactions of redox enzymes can be probed under fast turnover conditions. The PFIRE method builds upon the direct electrochemical control provided by the technique of PFE, which provides no direct structural information, and couples it to IR spectroscopy at a carbon electrode. The PFIRE approach thus adds chemical insight to the information available from electrochemistry alone and is highly suited to study of redox proteins and enzymes involved in small molecule binding and activation. In addition, PFIRE can provide information about potential-dependent structural changes in proteins in the absence of catalytic turnover. Such non-turnover electron transfer events are often difficult to detect using 'standard' applications of PFE, although the extension of PFE to Fourier-transformed AC voltammetry has been used with great success*.*^45^,^46^

The PFIRE method is, in principle, suitable for the study of any redox protein that can be studied using PFE. Therefore, as with PFE, protein adsorption is a critical step for a successful PFIRE experiment. In this protocol we describe an application of the PFIRE technique using *E. coli *Hyd1 as a case study.^36^,^43^ However, we have also applied the PFIRE technique to the cytoplasmic regulatory hydrogenase from *R. eutropha*,^44^ and to flavin mononucleotide adsorbed on carbon black.^40^ In all these cases, simple physical adsorption to unmodified high surface area carbon black (as described in this Protocol) provides a surface coverage of protein that is high enough to record good quality IR spectra with a high signal-to-noise ratio. In cases where such high levels of adsorption cannot be achieved it may be necessary to modify the surface of the carbon particles, for example to allow covalent attachment of protein to the electrode surface.^60^,^61^,^62^ The use of a glovebox for PFIRE measurements is only strictly necessary when studying samples that must be handled anaerobically. However in practice the extremely constant and low (< 80 °C dew point) levels of water vapor provided by the glovebox atmosphere give high signal-to-noise levels that allow the extraction of very small absorbances.^44^ In many cases an anaerobic environment, such as that provided by the glovebox, is also desirable for the electrochemical measurement (integral to the PFIRE technique) in order to avoid current due to O_2_ reduction at the working electrode.

IR absorbances due to bulk water, experimental buffers and the carbon particles on which the sample is adsorbed all contribute significantly to the experimental spectra and could overlap bands of interest, particularly in the amide I, II and III regions of the spectrum.^63^ The amide region also contains information from organic species such as flavins or nicotinamide cofactors, as well as the substrates and products of many oxidation and reduction reactions. In the case of NiFe hydrogenases, *ν*_CO_ and *ν*_CN_ bands of the active site fall in a relatively clear region of the spectrum and so the PFIRE technique is very well suited to the study of these enzymes. In other cases, however, difference spectra coupled with isotopic labelling approaches may be needed to isolate changes due to the immobilized protein. Similar approaches have been used to identify, for example, protonation changes, structural rearrangements and to study Michaelis-Menten complexes using IR spectroscopy.^64^,^65^,^66^ PFIRE is not, therefore, limited to the study of hydrogenases but can be applied to any redox protein that contains (or whose substrates, products or inhibitors contain) groups with diagnostic IR-active vibrations; carbon monoxide dehydrogeanses,^67^ nitrogenases,^68^,^14^ flavoproteins,^40^ and formate dehydrogenases, for example.

The related technique, SEIRA, is very well suited to the study of membrane-associated proteins in a biomimetic environment.^32^ SEIRA is an adaptation of IR spectroscopy that also uses an ATR-IR configuration, and makes use of a surface enhancement effect that amplifies the IR absorbance of molecules located close to (within a few nm) the surface of the ATR prism (IRE). SEIRA is therefore exquisitely sensitive to spectral changes that occur within the adsorbed protein and membrane architecture and is relatively free from competing signals from solvent and substrates/inhibitors present in solution. This is somewhat in contrast to the PFIRE technique described here which relies on a significantly greater penetration depth above the surface of the IRE (~1 µm), meaning that PFIRE is more sensitive to substrates, products or inhibitors present in solution. This increased sensitivity to 'bulk' solvent can be advantageous; if the substrate or product can be observed directly by IR, PFIRE spectra report on both steady state kinetics of long-lived active species and associated product formation during electrocatalysis.^69^ The ability to observe steady state concentrations of substrate and product will be particularly valuable for enzymes such as carbon monoxide dehydrogenase (which catalyzes the reversible oxidation of CO to CO_2_, a strong IR absorber) or formate dehydrogenase (which catalyzes the reversible oxidation of formate to CO_2_).

At present, PFIRE is limited to steady-state kinetic studies of enzyme electrocatalysis due to the macroscopic carbon electrodes used to 'concentrate' the enzyme onto the surface of an IRE for data collection in and ATR-IR geometry. In this respect PFIRE studies of NiFe hydrogenases are complementary to the work of Dyer and co-workers,^49^,^50^ who use light-triggered transient absorption IR spectroscopy to study sub-turnover kinetics. Work is underway to miniaturize the spectroelectrochemical cell,^40^ and with the use of microelectrodes time resolution on the order of microseconds should be achievable. This will enable study of sub-turnover kinetics for enzymes with turnover frequencies up to *ca* 100 - 500 s^-1^, and will enable the study of both reductive and oxidative processes.

Overall, PFIRE is a spectroscopic technique that allows chemical characterization of electrocatalytic reactions of redox enzymes under steady state conditions. The PFIRE approach allows multiple chemical and electrochemical titrations to be carried out on the same enzyme sample, as the high surface area electrodes used provide a robust adsorption of protein and the ATR-IR geometry allows facile solution exchange. The ability to collect such structural information *in situ* during enzyme function is an invaluable tool for the wider bioelectrochemistry community.

## Disclosures

The authors declare no competing financial interest.
